# Detecting Melanocortin 1 Receptor Gene’s SNPs by CRISPR/enAsCas12a

**DOI:** 10.3390/genes14020394

**Published:** 2023-02-02

**Authors:** Wei Yang, Dagang Tao, Bingrong Xu, Yueting Zheng, Shuhong Zhao

**Affiliations:** 1 Key Laboratory of Agricultural Animal Genetics, Breeding and Reproduction of Ministry of Education & Key Lab of Swine Genetics and Breeding of Ministry of Agriculture and Rural Affairs, Huazhong Agricultural University, Wuhan 430070, China; 2 Guangdong Laboratory of Lingnan Modern Agriculture, Guangzhou 510642, China; 3 Key Laboratory of Animal Biochemistry and Nutrition, Ministry of Agriculture and Rural Affairs and Key Laboratory of Animal Growth and Development of Henan Province, College of Veterinary Medicine, Henan Agricultural University, Zhengzhou 450046, China

**Keywords:** CRISPR/enAsCas12a, *MC1R*, SNP, molecular diagnostics

## Abstract

Beyond its powerful genome-editing capabilities, the CRISPR/Cas system has opened up a new era of molecular diagnostics due to its highly specific base recognition and trans-cleavage activity. However, most CRISPR/Cas detection systems are mainly used to detect nucleic acids of bacteria or viruses, while the application of single nucleotide polymorphism (SNP) detection is limited. The *MC1R* SNPs were investigated by CRISPR/en*As*Cas12a and are not limited to the protospacer adjacent motif (PAM) sequence in vitro. Specifically, we optimized the reaction conditions, which proved that the en*As*Cas12a has a preference for divalent magnesium ion (Mg^2+^) and can effectively distinguish the genes with a single base difference in the presence of Mg^2+^, and the Melanocortin l receptor (*MC1R*) gene with three kinds of SNP sites (T305C, T363C, and G727A) was quantitatively detected. Since the en*As*Cas12a is not limited by PAM sequence in vitro, the method shown here can extend this extraordinary CRISPR/en*As*Cas12a detection system to other SNP targets, thus providing a general SNP detection toolbox.

## 1. Introduction

The clustered regularly interspaced short palindromic repeats (CRISPR) and CRISPR-associated proteins (Cas) have been widely used in molecular diagnostics due to their simple operation and rapid detection system [1,2]. After the target DNA fragment is recognized by the CRISPR/Cas complex, the Cas is activated and randomly cleaves the ssDNA reporters with quenched fluorescence to release fluorescent signals. The crRNA is one of the most important parts of the CRISPR/Cas detection system, which includes two parts: the invariant skeleton, which is universal for each Cas, and the variable part, which differs according to the target gene sequence. The other important part of the CRISPR/Cas detection system is the protospacer adjacent motif (PAM) requirement, located near the target gene and affecting Cas’s endonuclease activity. Therefore, the molecular diagnostics of the CRISPR/Cas, by interacting with crRNAs, should enable the detection of any nucleic acid fragment in the genome in theory. However, the available target sequence is limited by the need for a specific PAM sequence. Until now, the CRISPR/Cas systems, as a new generation of detection methods, have been only used for detecting the sequence of various pathogens, such as SARS-CoV-2, Zika virus, MERS, Dengue virus, Japanese encephalitis virus (JEV), Porcine reproductive and respiratory syndrome virus (PRRSV), and African swine fever virus (ASFV) [3,4,5,6,7,8,9,10]. A series of studies have been carried out on designing and optimizing components, including crRNA, Cas, and the signal output of ssDNA, to make the detection more efficient and sensitive [11,12,13,14,15,16,17]. Although great progress has been made, almost all reported CRISPR/Cas systems are developed to detect the fragment of nucleic acid with PAM nearby. Therefore, more approaches are needed to develop and optimize the CRISPR/Cas system that can detect any SNP targets beyond the targets of nucleic acid fragments and is not limited to the specific PAM sequence.

Single nucleotide polymorphisms (SNPs) mainly refer to the polymorphisms in the DNA sequence caused by the variations of a single nucleotide at the genomic level. SNPs are widespread in the pig genome, including both genetic coding regions and non-coding regions. As the third-generation molecular marker, SNP is widely used in molecular genetics, forensic material evidence testing, disease diagnosis, and treatment. In addition, SNP detection is of great significance in genetics and breeding. At present, there are four main SNP detection methods: TaqMan probe detection and molecular beacon detection, which are based on probe specificity [18]; ARMS-PCR and KASP detection, which are based on primer specificity [19,20,21]; SNaPshot; and mass spectrometry methods which are based on single base extension technology and sequencing [22]. The main problems of the first two methods are complex designs and tedious processes; then, the latter two methods must be carried out by the professional organization and the need for professional analysis. To overcome these disadvantages, the CRISPR/Cas system, based on its recognition specificity and trans-cleavage activity, has been reported in detecting SNP, but the dependence on the PAM sequence limits its application. In addition, the enzyme activity of Cas could be affected by reaction conditions, and it has the best enzyme activity only in appropriate reaction conditions [23,24,25]. To achieve the general application of CRISPR/Cas in SNP detection, we herein report an engineered Cas12a from *Acidaminococcus* (en*As*Cas12a), which was used in the detection of the melanocortin 1 receptor (*MC1R*) gene with SNPs.

The *MC1R* is an important gene that controls melanin synthesis in animals [26]. It is expressed in melanocytes, adrenal cortical cells, the nervous system, and the immune system and has different functions [27,28,29]. The melanocortin receptor in melanocytes is called “melanocortin receptor 1 (MC1R)”. In mammals, the *MC1R* is the main gene regulating skin and hair color. In addition, the MC1R is also related to the formation of human cancer. With the extensive development of research on the hair color of different breeds of pigs, the *MC1R* gene of pigs has been a useful genetic marker, which can be used to identify the purity of breeds, and genetic relationships, determine cross combinations, and evaluate product quality [30,31,32]. It has been reported that the SNPs of the Porcine *MC1R* gene in T305C, T363C, and G727A are directly related to skin color traits (Table 1), and its identification is broadly used in genetic breeding. Here, we chose the CRISPR/en*As*Cas12a system because it has been shown that en*As*Cas12a has higher enzyme activity [13]. It is well known that CRISPR/Cas detection technology originates from CRISPR/Cas editing technology. Based on this principle, a reporter molecule with fluorescent and quenched groups is added to the system. When the CRISPR/Cas system recognizes the target sequence, the reporter sequence will be cut, and then the fluorescence signal generated by the fluorescent group will be measured to determine whether the target gene in the sample exists. The precise identification of the CRISPR/Cas detection system brings high specificity and high sensitivity to the detection, which makes CRISPR/Cas detection technology show great potential in molecular detection. By reacting with different crRNAs, we demonstrated that the PAM sequence is not strictly dependent when en*As*Cas12a recognizes the target nucleic acid sequence in vitro. Subsequently, through optimization of reaction conditions, the results showed that the enzyme activity of en*As*Cas was increased, and the CRISPR/en*As*Cas12a could distinguish between the *MC1R* gene and *MC1R* with SNPs. Altogether, the results confirmed that the CRISPR/en*As*Cas12a assay system holds the potential to avoid the strict dependence on PAM and simplify the detection processes that will be widely used in SNP detection.

## 2. Materials and Methods

### 2.1. The Expression and Purification of enAsCas12a and LbCas12a

The gene of en*As*Cas12a was synthesized in pUC (GenScript, Nanjing, China) and then cloned into pET28 with a 6His tag at C-terminal. The plasmid of pET28-enAsCas12a was transformed into Escherichia coli BL21 (DE3). A single clone was selected from a Kanamycin-resistant plate and inoculated into 5 mL LB medium for 12 h at 37 °C. Then, the bacterial solution was inoculated into 200 mL LB medium at the ratio of 1/1000 and Isopropyl β-D-1-thiogalactopyranoside (IPTG) was added with the final concentration of 0.5 mM when OD600 reached 0.6–0.8. The bacterial solution was continuously cultured for another 12 h at 18 °C and harvested.

The cell pellets were washed again and then dissolved in a lysis solution. After ultra-sonication, the en*As*Cas12a was purified by affinity purification using an Ni–NTA (Nickel-nitrilotriacetic acid) resin column (TransGen Biotech, Beijing, China). Then, the purified en*As*Cas12a was concentrated by centrifugation and dialyzed to remove imidazole. At last, the purified en*As*Cas12a was stored in a storage buffer (20 mM PBS, pH 7.5, 20 mM NaCl, 10% (*v/v*) glycerol) and quantified using the BCA Protein Assay Kit (TAKARA, Beijing, China). The results are shown in Supplementary Figure S1.

The gene of *Lachnospiraceae* Cas12a (*Lb*Cas12a) was synthesized in pUC (GenScript, Nanjing, China) and then cloned into pET28 with a 6His tag at C-terminal. The *Lb*Cas12a was expressed and purified in the same way.

### 2.2. The Selection of Target Genes of MC1R

We selected three fragments of the *MC1R* gene, each containing one SNP site (T305C, T363C, and G727A) (Table S1). The PCR reactions were conducted as the following conditions: initial preheating to activation of Taq polymerase at 96 °C for 2 minutes (min), followed at 96 °C for 2 min, 55 °C for 30 seconds (s) and 72 °C for 30 s with 30 cycles of denaturation, annealing, and extension, and then an extra extension at 72 °C for 3 min. The lengths of the amplificated fragment of the *MC1R*-305, *MC1R*-T305C, *MC1R*-363, *MC1R*-T363C, *MC1R*-727, and *MC1R*-A727G were 96 bp, 96 bp, 98 bp, 98 bp, 94 bp, and 94 bp, respectively. The PCR reaction system was carried out in a 50 µL reaction volume, including 25 µL 2 × Taq DNA polymerase Mix (TSINGKE, China), 1 µL of the forward primer (10 pmol), 1 µL of reverse primers (10 pmol), 1 μL of template DNA (1ng), and adding 22 µL of H_2_O to 50 µL. The PCR products were purified (PCR purification kit, Beijing Tianmo Sci & Tech Development, Beijing, China), and the concentration of PCR products were quantified with Nano drops 2000 spectrophotometer (Thermo Fisher Scientific, Wilmington, DE, USA). It was stored at −20 °C and used as the target gene for in vitro detection.

### 2.3. The RNA Transcription In Vitro

The skeleton sequence of crRNA was as reported (gggAATTTCTACTGTTGTAGAT). The skeleton sequence of crRNA and the fragment of the target gene were combined by overlap PCR using a forward primer with a T7 promoter and a reverse primer with the target sequence (Figure S2). The PCR products were purified with a PCR purification kit (Beijing Tianmo Sci & Tech Development, Beijing, China), and used as a transcription template for in vitro transcription.

To obtain the crRNA with a specific target gene, the products of PCR were used as the DNA templates for in vitro transcription (HiScribe T7 High Yield RNA Synthesis Kit, New England Biolabs, Beijing, China). To remove the DNA template, DNase enzyme was added for 15 min at 37 °C (Cat. No. M0303S, New England Biolabs, Beijing, China). In the transcription reaction, a 20 µL transcription reaction mixture containing 1 µL T7 polymerase (5 U µL ^−1^), 1 µL ATP (2.5 mM), 1 µL GTP (2.5 mM), 1 µL CTP (2.5 mM), 1 µL UTP (2.5 mM), 2 µL transcription buffer (10×), 5 µL target gene fragment (100 ng), and 8 µL H_2_O to the final volume. It was incubated at 37 °C for 8–10 h. Subsequently, the transcribed crRNA in vitro was purified (Monarch RNA Cleanup Kit, New England Biolabs, Beijing, China), and the concentration of crRNA was quantified with Nano drops 2000 spectrophotometer (Thermo Fisher Scientific, Wilmington, DE, USA). The copy numbers of crRNA were calculated by their concentration and length.

### 2.4. Optimization of the CRISPR/enAsCas12a Detection Assay

First, we optimized the concentration of PBS because the reaction buffer significantly affects the enzyme activity. The CRISPR/en*As*Cas12a reaction volume was 20 µL containing 1 × 10^−3^ mM crRNA-305-3, 200 ng en*As*Cas12a, 20 × 10^−9^ mM ssDNA reporter, 2 µL 10 × NEB buffer 2.1, 1 µL the target gene (20 ng/µL, *MC1R*-305), PBS buffer (10, 20, 50, and 100 mM), and H_2_O added to the final volume. The reaction mixture was incubated at 37 °C for 10 min, and the reaction was terminated by incubation at 95 °C for 5 min. Then, the 20 mM PBS with different concentration Mg^2+^ (0, 5, 10, 20, 30, 40, 50, 60, 70, 80, and 90 mM MgCl_2_) was tested.

### 2.5. The CRISPR/enAsCas12a Detect the MC1R Gene and the MC1R Gene with SNP

The single-strand DNA reporter with the fluorescent group at 5′ end and quenching group at 3′ ends (ssDNA-FQ), namely ROX-N12-BHQ2, was synthesized (TSINGKE, Beijing, China) and was listed in the Supplementary Table S2. The CRISPR/en*As*Cas12a detection assays were carried out as described in the previous report [3] and slightly optimized. In a 20 µL reaction system, it contained 200 ng purified en*As*Cas12a, 20 × 10^−9^ mM ssDNA with quenched fluorescent group sensor, 1 × 10^−3^ mM purified crRNA, and 1 µL of the target gene (*MC1R*-gene or *MC1R*-SNP gene) in the reaction buffer (20 mM PBS, 20 mM NaCl, 40 mM MgCl_2_, pH 7.4) and the reaction was incubated at 37 °C for 10 min. The reaction was terminated by heating at 95 °C for 10 min. For the fluorescence detection, the absorbance is 585 nm, and the emission is 605 nm.

### 2.6. Statistical Analysis

Statistical analysis of the florescent signals was performed using GraphPad Prism 8. The mean ± (SEM) standard error was obtained from every reaction sample of three independent experiments. The two-tailed Student’s *t*-test was used to compare significant differences between the two reaction samples (**** *p* < 0.0001, *** *p* < 0.001, ns: not significant at the 95% confidence level).

## 3. Results

### 3.1. The Detection of the MC1R Gene by CRISPR/enAsCas12a System

**Figure 1:**
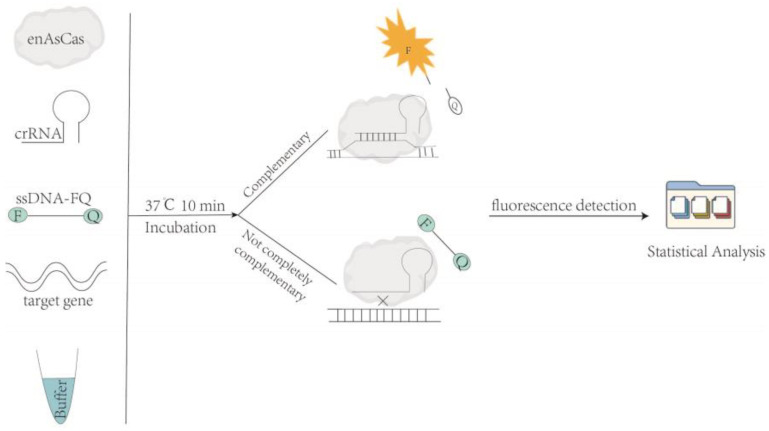
The CRISPR/en*As*Cas12a assay system in vitro.

As shown in Figure 1, the CRISPR/enAsCas12a assay system is prepared by adding expressed and purified en*As*Cas12a, transcribed and purified crRNA, single-stranded DNA reporter with fluorophore and quencher (ssDNA-FQ), and the synthesized target gene. After incubating the CRISPR/en*As*Cas12a reaction system at 37 °C for 10 min, the reaction was terminated at 95 °C for 10 min. If the crRNA of the en*As*Cas12a-crRNA complex is complementary to the target gene, the trans-endonuclease of en*As*Cas12a is activated, which then cuts the ssDNA-FQ and generates a fluorescent signal. Otherwise, if the crRNA of the en*As*Cas12a-crRNA complex is not completely complementary to the target gene, the enAsCas12a could not be activated to produce a fluorescent signal.

**Figure 2:**
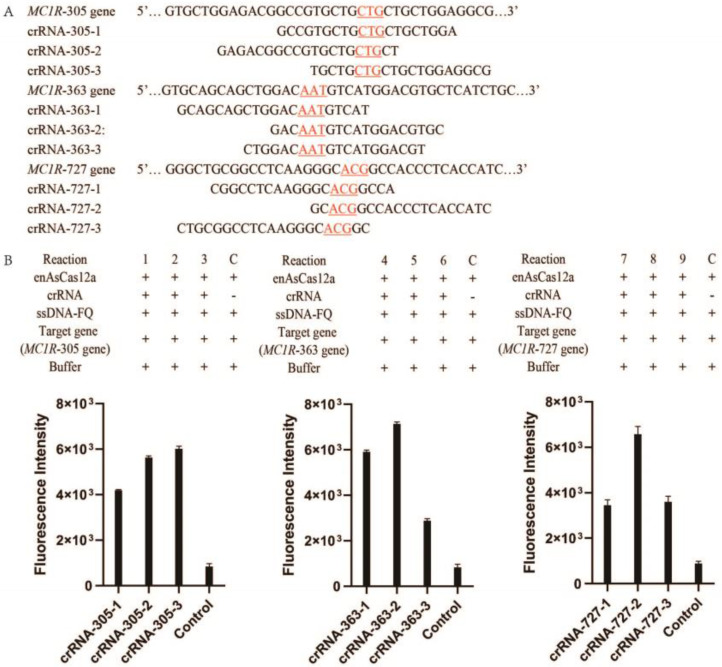
The *MC1R* detection by the CRISPR/en*As*Cas12a. (**A**). The *MC1R* gene fragment containing 305, 363, and 727 site; the variable part of the crRNA sequence (the red parts translate to the 102nd, the 121st, and the 243rd amino acid of MC1R); (**B**). the fluorescence detection of every reaction (C, control).

For a detailed analysis of the CRISPR/en*As*Cas12a assay system, we designed and detected nine reaction systems with various crRNAs (reactions 1–9). Nine crRNA without PAM limitation were selected according to the *MC1R* gene fragments. The sequence of *MC1R* gene fragments and the various part of crRNAs are listed (Figure 2A). The *MC1R* gene was also synthesized (Table S1). In theory, the reaction without crRNA was used as the negative control and the background, and it shows that there is no background interference fluorescence and no generation of fluorescence. The reactions with crRNA, which have different a variable part, are tested and compared.

The results showed that each crRNA without additional PAM could combine with en*As*Cas12a and activate its trans-cleavage activity to detect specific genes even though it has different effects on Cas activity (Figure 2B). In Figure 2B, the results also showed that the fluorescence intensity of reaction 3 is better than reaction 1 and reaction 2, the fluorescence intensity of reaction 5 is better than reaction 4 and reaction 6, and the fluorescence intensity of reaction 8 is better than reaction 7 and reaction 9. *Lb*Cas12a, commonly used in molecular diagnostics in vitro, was also tested in the same way and could not be activated by crRNA without PAM (The results were shown in Supplementary Materials). Therefore, the results confirmed that the activity of en*As*Cas12a is mainly dependent on the crRNA and the target gene.

### 3.2. Optimization of the CRISPR/enAsCas12a Assay System

**Figure 3:**
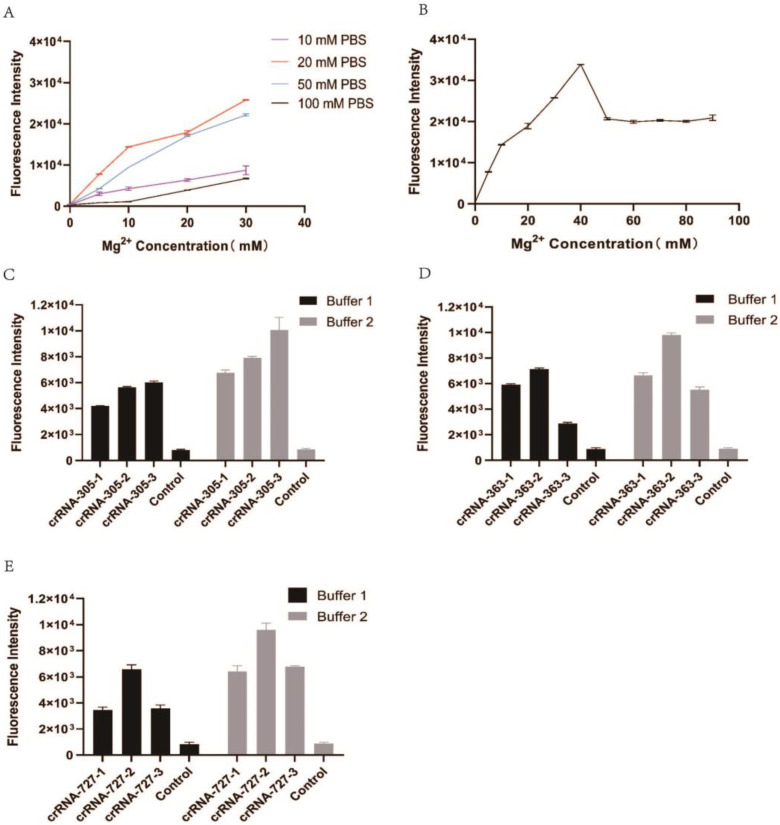
Optimization of the CRISPR/en*As*Cas12a assay system. (**A**) en*As*Cas12a-mediated *MC1R* detection in 10, 20, 50, and 100 mM PBS buffer with different concentrations of Mg^2+^; (**B**) en*As*Cas12a-mediated *MC1R* detection in 20 mM PBS buffer with different concentrations of Mg^2+^; (**C**–**E**). en*As*Cas12a-mediated nucleic acid fragment detection of *MC1R* (containing 102, 121, and 243 site) in different buffer solutions (Buffer1: 20 mM PBS, 10 mM Mg^2+^; Buffer2: 20 mM PBS, 40 mM Mg^2+^).

It is well known that buffer solution and metal ions affect enzyme activity. We aimed to optimize the solution conditions and Mg^2+^ concentration in the CRISPR/en*As*Cas12a assay system. First, we tested four different concentrations of phosphate buffer saline (PBS, 10, 20, 50, and 100 mM) that affect the sensitivity of en*As*Cas12a in nucleic acid detection. We found that en*As*Cas12a-mediated nucleic acid detection has the highest reaction efficiency in 20 mM PBS buffer compared with 10, 50, and 100 mM PBS (Figure 3A). Then, we screened divalent Mg^2+^ for their effect on the activity of en*As*Cas12a. The results showed that en*As*Cas12a is a kind of enzyme with strict metal ion dependence, and its activity is the best at the concentration of 40 mM Mg^2+^, rather than the commonly used concentration of 10 mM Mg^2+^ (Figure 3B).

To further confirm the influence of Mg^2+^ on the CRISPR/en*As*Cas12a assay system, the reaction efficiency of the nine reaction systems (reactions # 1–9) was tested in the 20 mM PBS with 10 or 40 mM Mg^2+^. The results showed that the fluorescence intensity of all reactions is improved more in 40 mM Mg^2+^ than in 10 mM Mg^2+^ (Figure 3C–E). Therefore, 40 mM Mg^2+^ enables higher sensitivity to the CRISPR/en*As*Cas12a assay in 20 mM PBS.

### 3.3. The SNPs of MC1R Detection by CRISPR/enAsCas12a

It has been reported that three kinds of SNPs (T305C, T363C, and G727A) of the *MC1R* gene affect skin color directly. The *MC1R* gene fragments with the SNP site were synthesized: the first fragment containing the T305C site (*MC1R*-T305C gene), the second fragment containing the T363C site (*MC1R*-T363C gene), and the third fragment containing the A727G site (*MC1R*-A727G gene). The corresponding *MC1R* gene fragments were also synthesized (Table 2). Here, we evaluated *MC1R* SNPs’ detection using the CRISPR/en*As*Cas12a assay system. The crRNA-305-3, crRNA-363-2, and crRNA-727-2, which had promoted the activity of enAsCas12a according to the previous results (Figure 2B), were selected for further detection. Each crRNA is complementary to the *MC1R* gene while not completely complementary to the *MC1R* gene with SNP.

**Figure 4:**
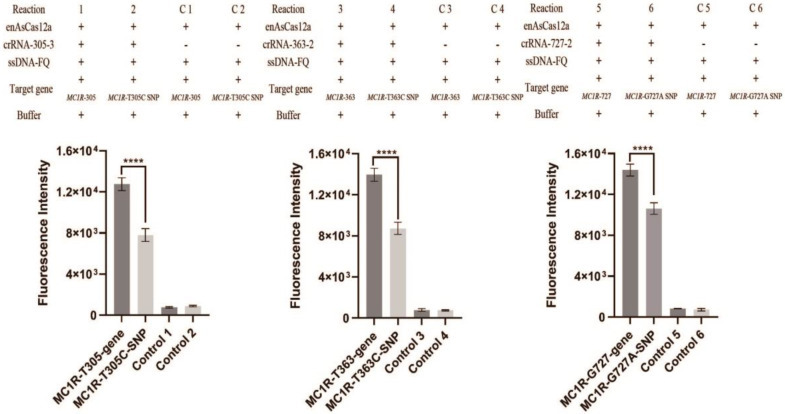
*MC1R* SNPs detection by CRISPR/enAsCas12a assay system. The CRISPR/en*As*Cas12a assay system to detect *MC1R*-305-gene fragment and *MC1R*-T305C-SNP gene fragment in vitro (the left panel); the CRISPR/en*As*Cas12a assay system to detect *MC1R*-363-gene fragment and *MC1R*-T363C-SNP gene fragment in vitro (the middle panel); the CRISPR/en*As*Cas12a assay system to detect *MC1R*-727-gene fragment and *MC1R*-G727A-SNP gene fragment in vitro (the right panel) (**** *p* < 0.0001).

In Figure 4, the reaction 1 (R 1) and the negative control 1 (C 1) have the same target gene (*MC1R*-305 gene), while C 1 is the negative control without addition of crRNA. Similarly, the reaction 2 (R 2) and the negative control 2 (C 2) have the same target gene (*MC1R*-T305C gene), the reaction 3 (R 3) and the negative control 3 (C 3) have the same target gene (*MC1R*-363 gene), the reaction 4 (R 4) and the negative control 4 (C 4) have the same target gene (*MC1R*-T363C gene), the reaction 5 (R 5) and the negative control 5 (C 5) have the same target gene (*MC1R*-727 gene), and the reaction 6 (R 6) and the negative control 6 (C 6) have the same target gene (*MC1R*-A727G gene). The results showed that all the negative controls have weak fluorescence as the background. The results also showed that the fluorescence intensity of reaction 1 is significantly higher than reaction 2 (Figure 4, left panel), the fluorescence intensity of reaction 3 is significantly higher than reaction 4 (Figure 4, middle panel), and the fluorescence intensity of reaction 5 is significantly higher than reaction 6 (Figure 4, right panel). Therefore, the CRISPR/en*As*Cas12a assay system can distinguish between the *MC1R* gene and the *MC1R* gene with an SNP site, which has only one base difference.

## 4. Discussion

The MC1R, a key factor in melanogenesis synthesis, is the main factor affecting hair and skin color and a major determinant of sun sensitivity. It has been reported that MC1R affects animals’ skin and fur color by controlling the synthesis of true melanin. It is also associated with many important production properties and affects the occurrence of many diseases. In addition, the SNPs of the *MC1R* gene can be used as an important indicator to determine the purity of varieties, breeds, and other studies, so the studies on skin and fur color and the related SNP detection have received wide attention. Many techniques for detecting SNPs have been established (as described above), but these methods cannot be widely used due to their complex operation and high technical requirements.

CRISPR/Cas diagnosis has received extensive attention in the past few years due to its accuracy, sensitivity, and rapidity. Here we used CRISPR/en*As*Cas12a, which is optimized to improve its detection efficiency, to detect the SNPs of the *MC1R* gene. To detect the *MC1R* gene, nine random crRNA, without adding additional PAM sequences, were designed, and the variable region of the crRNA is complementary to the *MC1R* gene fragments (Figure 2A). The results showed that every crRNA could activate the trans-exonuclease activity of en*As*Cas12a and the strong fluorescence could be produced (Figure 2B). So, unlike other Cas proteins, en*As*Cas12a could be activated by crRNA independently on specific PAM sequences in vitro and detect any gene fragments without PAM. We also found that CRISPR/en*As*Cas12a can detect all target nucleic acid sequences without PAM, but its detection efficiency is different due to the variable sequence contained in crRNA. These results confirmed that the activity of en*As*Cas12a is mainly affected by the target gene sequence. Therefore, we speculate that en*As*Cas12a has some base preference, which requires further confirmation. Furthermore, we optimized the solution buffer and the concentration of divalent Mg^2+^ and found that the CRISPR/en*As*Cas12a assay system is more sensitive in 20 mM PBS with 40 mM Mg^2+^. Finally, we proved that the CRISPR/en*As*Cas12a assay system could distinguish between genes and SNPs with a single base nucleotide difference. So, the current approach confirms the detection activity of CRISPR/*enAs*Cas*12a* and the sensitivity for detecting SNPs without PAM, thus expanding the application of CRISPR/en*As*Cas12a in vitro detection and eliminating the limitation of PAM.

In summary, this method has more advantages than traditional technologies. First, the costs are significantly decreased. If the samples to be tested are sent to the relevant testing organization or company, each sample will cost from ten to tens of dollars, while the method we have established needs only about USD 1. So, this is a low-cost diagnostic analysis with high sensitivity. Second, this method is fast. The whole process of SNP detection based on CRISPR/Cas needs about 1–2 h, while other detection methods require large instruments and equipment, professional testing institutions, or companies, and the samples must be transported. We have to wait at least one day to receive the results; this method is rapid in detection. Third, it is an easy diagnostic approach. If the reagents for detection are provided, they can be carried out in general laboratories. Thus, this technology is a low-cost, convenient, and rapid detection method.

## Figures and Tables

**Table 1:** The dominant SNP sites of the *MC1R* gene.

Allete	Coat Color	Codon		
		102	121	243
MC1R*1(*E*^+^)	Wild type, European wild boar	CTG Leu	AAT Asn	GCG Ala
MC1R*1(*E*^+^)	Wild type, Japanese wild boar	--- -	--C -	--- -
MC1R*2(*E^D1^*)	Dominant black	-C- -	--C -	--A -
MC1R*4(*e*)	Recessive red	--- -	--- -	A-- Thr

*E*, the *Extension* locus in pig; *E*^+^, wild type; ED, the dominant black; *e*, the recessive red. The dash (-) indicates the identity to the top base or amino acid.

**Table 2:** The sequence of the MC1R gene and its SNPs’ sequence.

Gene	Sequence
*MC1R*-305 gene	…5’-GAGACGGCCGTGCTGCTGCTGCTGGAGGCGGGC-3’…
*MC1R*-T305C SNP	…5’-GAGACGGCCGTGCTGCcGCTGCTGGAGGCGGGC-3’…
*MC1R*-363 gene	…5’-GTGCAGCAGCTGGACAATGTCATGGACGTGCTC-3’…
*MC1R*-T363C SNP	…5’-GTGCAGCAGCTGGACAAcGTCATGGACGTGCTC-3’…
*MC1R*-727 gene	…5’-TGCGGCCTCAAGGGCACGGCCACCCTCACCATC-3’…
*MC1R*-A727G SNP	…5’-TGCGGCCTCAAGGGCgCGGCCACCCTCACCATC -3’…

## Data Availability

Not applicable.

## Supplementary Materials

The following supporting information can be downloaded at: https://www.mdpi.com/article/10.3390/genes14020394/s1, Figure S1: The expression and purification of en*As*Cas12a and *Lb*Cas12a; Figure S2: The amplification results of crRNA (crRNA-102-1/2/3, crRNA-121-1/2/3, and crRNA-243-1/2/3); Figure S3: The *MC1R* detection by the CRISPR/*Lb*Cas12a; Table S1: the gene of *MC1R*, the fragment of *MC1R*-102/121 gene, and the fragment of *MC1R*-243 gene; Table S2: the sequence of ssDNA-FQ.
