# Supplementary material for: Detecting Melanocortin 1 Receptor Gene’s SNPs by CRISPR/enAsCas12a
Source: Genes (Basel). 2023 Feb 2;14(2):394. doi: 10.3390/genes14020394 (PMC9957350; doi:10.3390/genes14020394)
Supplement: Supplementary file 1 [file genes-14-00394-s001.zip › genes-2160444-supplementary.pdf]

Detecting Melanocortin l receptor gene's SNPs by CRISPR/en*As*Cas12a

Table of Contents

Table S1. The *MC1R* gene and the fragments of the *MCIR* and the *MC1R*-SNP
Table S2. The sequence of ssDNS-FQ (ROX-N12-BHQ2)
Fig. S1. The expression and purification of the en*As*Cas12a and the *Lb*Cas12a.
Fig. S2. The amplification and purification results of crRNA
Fig. S3. The *MC1R* detection by the CRISPR/*Lb*Cas12a.

Table S1. The *MC1R* gene and the fragments of the *MCIR* and the *MC1R*-SNP

**Name**	**Sequence**
The *MC1R* gene: >NC_010448.4:c182187-181225 *MC1R* [organism=Sus scrofa] [GeneID=494018]	ATGCCTGTGCTTGGCCCGGAGAGGAGGCTGCTGGCTTCCCT<br>CAGCTCCGCGCCCCCAGCCGCCCCCCGCCTCGGGCTGGCCG<br>CCAACCAGACCAACCAGACGGGCCCCCAGTGCCTGGAGGT<br>GTCCATTCCCGACGGGCTCTTCCTCAGCCTGGGGCTGGTGA<br>GCCTCGTGGAGAACGTGCTGGTGGTGGCCGCCATCGCCAAG<br>AACCGCAACCTGCACTCGCCCATGTACTACTTCGTCTGCTGC<br>CTGGCCGTGTCGGACCTGCTGGTGAGCGTGAGCAACGTGCT<br>GGAGACGGCCGTGCTGCTGCTGCTGGAGGCGGGCGCCCTG<br>GCCGCCCAGGCCGCCGTGGTGCAGCAGCTGGACAATGTCAT<br>GGACGTGCTCATCTGCGGCTCCATGGTGTCCAGCCTCTGCTT<br>CCTGGGCGCCATCGCCGTGGACCGCTACGTGTCCATCTTCTA<br>CGCGCTGCGCTACCACAGCATCGTGACGCTGCCCCGCGTGG

	GGCGGGCCATCGCGGCCATCTGGGCGGGCAGCGTGCTCTCC AGCACCCTCTTCATCGCCTACTACCACCACACGGCCGTCCTG CTGGGCCTCGTCAGCTTCTTCGTGGCCATGCTGGCGCTCATG GCGGTACTGTACGTCCACATGCTGGCCCGGGCCTGCCAGCA CGGCCGGCACATCGCCCGGCTCCACAAGACGCAGCACCCCA CCCGCCAGGGCTGCGGCCTCAAGGGCACGGCCACCCTCACC ATCCTGCTGGGCGTCTTCCTCCTCTGCTGGGCACCCTTCTTC CTGCACCTCTCCCTCGTCGTCCTCTGCCCCCAGCACCCCACC TGCGGCTGCGTCTTCAAGAACGTCAACCTCTTTCTGGCCCTC GTCATCTGCAACTCCATCGTGGACCCCCTCATCTACGCCTTC CGCAGCCAGGAGCTCCGCAAGACCCTCCAGGAGGTGCTGC AGTGCTCCTGGTGA
MC1R-gene (305,363)	5'-GTGCTGGAGACGGCCGTGCTGCTG…AATGTCATGGACGT GCTCATCTGCGGCTC-3'
MC1R-SNP (T305C, T363C)	5'-GTGCTGGAGACGGCCGTGCTGCCG…AACGTCATGGACGT GCTCATCTGCGGCTC-3'
MC1R-gene (243)	5'-CAGGGCTGCGGCCTCAAGGGCACGGCCACCCTCACCATC CTG-3'
MC1R-SNP (G727A)	5'-CAGGGCTGCGGCCTCAAGGGCGCGGCCACCCTCACCATC CTG-3'
MC1R-L102 -F:	GACCTGCTGGTGAGCGTGAGCAACGTGCTGGAGACGGCCG TGCTGCTGCTGCTGGAG
MC1R-L102 -R:	CTGCTGCACCACGGCGGCCTGGGCGGCCAGGGCGCCCGCCT CCAGCAGCAGCAGCAC
MC1R-A243 -F:	GACGCAGCACCCCACCCGCCAGGGCTGCGGCCTCAAGGGC GCGGCCACCCT
MC1R-A243 -R:	CAGCAGAGGAGGAAGACGCCCAGCAGGATGGTGAGGGTGG CCGCGCCCTTGAGGC
MC1R-N121 /V122-F:	GCGCCCTGGCCGCCCAGGCCGCCGTGGTGCAGCAGCTGGAC AATGTCATGGACGTGCT
MC1R-N121 /V122-R:	CAGGAAGCAGAGGCTGGACACCATGGAGCCGCAGATGAGC ACGTCCATGACATTGTCCA

Table S2. The sequence of ssDNS-FQ (ROX-N12-BHQ2)

Name	Sequence
ssDNA-FQ	5'-ROX/GTATCCAGTGCG/3'-BHQ2

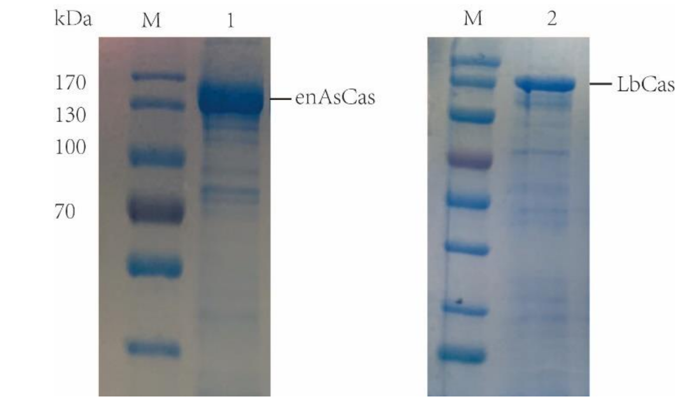

Figure S1. The expression and purification of the en*As*Cas12a and the *Lb*Cas12a.

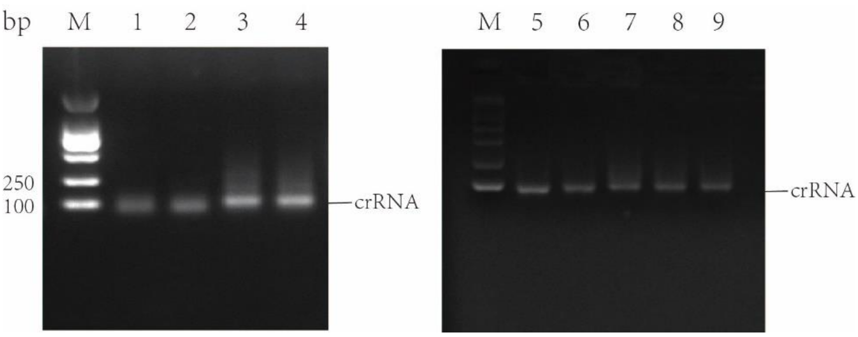

Figure S2. The amplification and purification results of crRNA

M, DNA marker; 1, crRNA-305-1; 2, crRNA-305-2; 3, crRNA-305-3; 4, crRNA-363-1; 5, crRNA-363-2; 6, crRNA-363-3; 7, crRNA-727-1; 8, crRNA-727-2; 9, crRNA-727-3.

Reaction	1	2	3	C
*Lb*Cas12a	+	+	+	+
crRNA	+	+	+	-
ssDNA-FQ	+	+	+	+
Target gene (*MC1R*-305 gene)	+	+	+	+
Buffer	+	+	+	+

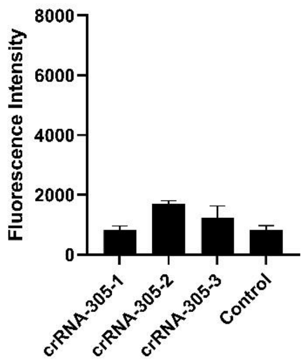

Figure S3. The *MC1R* detection by the CRISPR/*Lb*Cas12a.

Reaction 1, crRNA-305-1; Reaction 2, crRNA-305-2; Reaction 3, crRNA-305-3; Control, no crRNA
